# Addressing health disparities through implementation science—a need to integrate an equity lens from the outset

**DOI:** 10.1186/s13012-022-01189-5

**Published:** 2022-01-31

**Authors:** Andrew D. Kerkhoff, Erica Farrand, Carina Marquez, Adithya Cattamanchi, Margaret A. Handley

**Affiliations:** 1 Division of HIV, Infectious Diseases and Global Medicine, Department of Medicine, University of California San Francisco, San Francisco, CA USA; 2 Division of Pulmonary and Critical Care Medicine, Department of Medicine, University of California San Francisco, San Francisco, CA USA; 3 Partnerships for Research in Implementation Science for Equity Center, University of California San Francisco, San Francisco, CA USA; 4 Department of Epidemiology and Biostatistics, University of California San Francisco, San Francisco, CA USA

**Keywords:** Equity, Disparities, Community engagement, Co-design, Implementation science

## Abstract

There is increasing attention being given to opportunities and approaches to advance health equity using implementation science. To reduce disparities in health, it is crucial that an equity lens is integrated from the earliest stages of the implementation process. In this paper, we outline four key pre-implementation steps and associated questions for implementation researchers to consider that may help guide selection and design of interventions and associated implementation strategies that are most likely to reach and be effective in reducing health disparities among vulnerable persons and communities.

## Main text

In their article, Brownson and colleagues [1] identify three key challenges to advancing health equity through implementation science—(1) limitations of the current evidence base, (2) underdeveloped measures and methods, and (3) inadequate attention to context—and outline ten important action steps to address them. We support their concrete recommendations and those recently put forth by others [2–7] to better incorporate an equity lens into implementation science. In this paper, we seek to highlight not only the need to better incorporate equity into implementation science methods and frameworks, but also to have an equity focus from the outset of all implementation activities. Herein, we propose four pre-implementation planning steps and associated guiding questions (Table 1) that have been adapted from the early phases of the Knowledge-to-Action Framework [8] that we believe can elevate health equity throughout all processes represented by implementation science activities and complement the recommendations outlined by Brownson and colleagues [1].

1. *Identify important stakeholders related to equity and establish roles for partners throughout the entire implementation process*. Applying the central principles of research models for co-creation (i.e., community-based participatory research and integrated knowledge translation [9]), stakeholders across all levels and sectors with a strong interest in the priority health problem being addressed should be involved in implementation planning; this requires thinking broadly and giving the stakeholder engagement process adequate time. There should be strong attention to the meaningful involvement of individuals from and representing vulnerable populations (communities disproportionately affected by health inequities, including racial/ethnic minorities, socioeconomically disadvantaged communities, sexual and gender minorities, and indigenous peoples, among others [10]), acknowledging that such persons may be unable or unwilling to participate in all stages of the implementation process and should not be excluded on this basis [11]. Preferences for how and when to participate in the implementation process should be elicited, and where possible, flexibility and choices for multiple involvement opportunities should be provided. In particular, forging community partnerships can give voice to vulnerable populations, facilitate cross-sector collaborations and encourage synergies between communities and researchers, programmers, and/or policymakers [10]. Further, this stage should include developing a plan that outlines and establishes an agreement for how partners, including stakeholders representing vulnerable populations, will be involved at all stages of the implementation and/or research process, and not only at the outset or at the end of a project, and how they will be compensated.
2. *Include equity-related considerations when deciding which intervention(s) to implement and de-implement*. Ideally, interventions should be co-created with community groups and other stakeholders to ensure that they are maximally aligned with community needs, available resources, and local context, rather than later being adapted for local relevance. When choosing from among several potential interventions to address a priority health problem, the strength of evidence for effectiveness must be considered, including whether this evidence is similarly robust across populations and settings. Some interventions have demonstrated effectiveness at reducing health disparities and should be prioritized for implementation when possible [2]. However, due to their exclusion in clinical studies, the external validity of some interventions among vulnerable persons and communities may be underdeveloped or unclear. If this is the case, then further pre-implementation research in conjunction with communities may be needed to develop an intervention that works for them (e.g., knowledge creation) [8, 12]. In addition to having lower levels of access and uptake of effective interventions, vulnerable populations may also be more likely to receive low-value interventions (e.g., those that provide no benefits or the risk/harms outweigh the benefits) [3]. This too can propagate health disparities, and thus, it is important to assess whether there are existing low-value interventions that should be prioritized for de-implementation [3]. At this stage, trusted messengers for vulnerable populations should also be identified and involved in building trust and support around an intervention.
3. *Evaluate the performance gap related to the intervention or program of interest in vulnerable populations*. The performance gap (i.e., the difference between current and ideal uptake of an intervention) and outcome gap (i.e., the expected improvement in outcomes including health disparities) should be assessed among vulnerable populations [13]. This will help determine how much potential there is to reduce health disparities related to quality outcomes—effectiveness, efficiency, patient-centeredness, safety, timeliness—through improved access to/uptake of an intervention. Steps 2 and 3 should be undertaken concurrently, as outcome gaps should be discussed with community members and stakeholders to inform selection of an intervention from among several possible options, in conjunction with other factors described above.
4. *Identify and prioritize barriers faced by vulnerable populations—including structural racism and power dynamics*. Vulnerable populations face unique individual-, health systems-, and community-level barriers to care, which differ across settings. Thus, it is crucial to undertake formative research involving those with relevant lived experiences and in conjunction with community partners and other stakeholders to identify which contextually specific barriers to accessing or receiving an intervention may be the most important ones to target. This should include assessing how historical and structural racism and power dynamics have and may continue to influence the implementation context [6, 14]. Multi-level implementation strategies to address the key barriers, again with specific attention to mitigating effects of structural racism and differential power dynamics, should be co-designed with community groups and other implementing partners. Implementation strategies shown to be effective at reducing inequities in health should be prioritized for integration into the design of multi-component strategies and tailored to the needs of vulnerable populations [15]. Strategies for reaching vulnerable populations should also be person-centered and community-focused—accounting for specific preferences when known [16, 17]—and incorporate “low-barrier” approaches. Finally, it is important that stakeholders are involved in defining indicators to be evaluated to ensure that the outcomes assessed and that define programmatic success are relevant and meaningful to vulnerable populations in a specific setting.

**Table 1:** Four steps and associated guiding questions to explore prior to implementation of interventions that can improve health equity throughout early phases of implementation science activities

Pre-implementation steps to promote equity in implementation research	Guiding questions for consideration
**Identify important stakeholders related to equity and establish roles for partners throughout the implementation process**	• Who are the key stakeholders for health problems among vulnerable populations in your setting/context? • What are stakeholders’, including vulnerable persons and community members, preferences for how to be involved throughout different phases of the implementation process? • Can choices be provided for different approaches to and opportunities for involvement across different implementation phases? • Have ways to ensure that stakeholders are appropriately compensated for their time been considered? • What are the priorities of stakeholders that align with or are at odds with implementation, and how can they be leveraged/addressed? • Has a plan been outlined with and agreed to by community members and other stakeholders about engagement processes across the implementation stages, including dissemination strategies?
**Include equity-related considerations when deciding which intervention(s) to implement and/or de-implement**	• Is the health problem you are interested in targeting a priority to vulnerable populations in your setting/context? • Does the intervention(s) being considered have strong potential to improve that health problem for vulnerable populations in your setting/context? • Is there an existing intervention related to that health problem that provides low-value to vulnerable populations in your setting/context that should be de-implemented? • Who are trusted messengers and sources for vulnerable populations in your setting/context?
**Evaluate the performance gap related to the intervention or program of interest in vulnerable populations**	• What is the performance gap for an intervention in vulnerable populations in your setting/context? • What is the outcome gap for an intervention in vulnerable populations in your setting/context? • Do the performance and outcome gaps differ across populations and settings?
**Identify and prioritize barriers faced by vulnerable populations—including structural racism and power dynamics**	• Have the individual-, health systems-, and community-level barriers that explain the performance gap among vulnerable populations in your setting/context been explored? • Have the social determinants of health among vulnerable populations in your setting/context (including historical and structural racism and power dynamics) been discussed with stakeholders to understand their importance and potential impact on the health problem, intervention and implementation strategies being considered? • What implementation strategies may overcome key barriers—including social determinants of health—and optimize reach among vulnerable populations in your setting/context? • Are outcomes that are important and meaningful to stakeholders, including vulnerable populations, known, and included in the evaluation plan? • What evaluation measures will allow you to ensure that your implementation strategies are improving (not worsening) health disparities among vulnerable populations in your setting/context?

To advance health equity using implementation science, it is vital that an equity focus is integrated into the earliest stages of the implementation process. Using the above steps as a guide during the pre-implementation stage may help to select interventions and associated implementation strategies that are most likely to reach and be effective among vulnerable persons and reduce health inequities across diverse communities and settings.

## Data Availability

N/A.

## References

[1] Brownson RC, Kumanyika SK, Kreuter MW, Haire-Joshu D (2021). Implementation science should give higher priority to health equity. Implement Sci.

[2] Baumann AA, Cabassa LJ (2020). Reframing implementation science to address inequities in healthcare delivery. BMC Health Serv Res.

[3] Helfrich CD, Hartmann CW, Parikh TJ, Au DH (2019). Promoting health equity through de-implementation research. Ethn Dis.

[4] Woodward EN, Matthieu MM, Uchendu US, Rogal S, Kirchner JE (2019). The health equity implementation framework: proposal and preliminary study of hepatitis C virus treatment. Implement Sci.

[5] Galaviz KI, Breland JY, Sanders M, Breathett K, Cerezo A, Gil O (2020). Implementation science to address health disparities during the coronavirus pandemic. Heal Equity.

[6] Shelton RC, Adsul P, Oh A, Moise N, Griffith DM (2021). Application of an antiracism lens in the field of implementation science (IS): recommendations for reframing implementation research with a focus on justice and racial equity. Implement Res Pract.

[7] Snell-Rood C, Jaramillo ET, Hamilton AB, Raskin SE, Nicosia FM, Willging C (2021). Advancing health equity through a theoretically critical implementation science. Transl Behav Med.

[8] Graham ID, Logan J, Harrison MB, Straus SE, Tetroe J, Caswell W (2006). Lost in knowledge translation&colon; Time for a map&quest. J Contin Educ Health.

[9] Nguyen T, Graham ID, Mrklas KJ, Bowen S, Cargo M, Estabrooks CA (2020). How does integrated knowledge translation (IKT) compare to other collaborative research approaches to generating and translating knowledge? Learning from experts in the field. Health Res Policy Sy.

[10] Fields J, Gutierrez RJ, Marquez C, Rhoads K, Kushel M, Fernandez A, et al. Community-academic partnerships to address COVID-19 inequities: lessons from the San Francisco Bay Area. NEJM Catal. 2021. Available from: https://catalyst.nejm.org/doi/pdf/10.1056/CAT.21.0135. Accessed 11 Jan 2022.

[11] O’Hara JK, Lawton RJ (2016). At a crossroads? Key challenges and future opportunities for patient involvement in patient safety. BMJ Qual Saf.

[12] Callejas Jr. LM. GP, Limon FJ. Bringing equity to implementation supplement community-defined evidence as a framework for equitable Implementation. Stanford Social Innovation Review [Internet]. Available from: https://ssir.org/articles/entry/community_defined_evidence_as_a_framework_for_equitable_implementation. Accessed 11 Jan 2022.

[13] Handley MA, Gorukanti A, Cattamanchi A (2016). Strategies for implementing implementation science: a methodological overview. Emerg Med J.

[14] Loper A, Woo B, Metz A. Equity is fundamental to implementation science. Stanford Social Innovation Review [Internet]. Available from: https://ssir.org/articles/entry/equitable_implementation_at_work. Accessed 11 Jan 2022.

[15] Gaias LM, Arnold KT, Liu FF, Pullmann MD, Duong MT, Lyon AR. Adapting strategies to promote implementation reach and equity (ASPIRE) in school mental health services. Psychol Sch. 2021.

[16] Beres LK, Simbeza S, Holmes CB, Mwamba C, Mukamba N, Sharma A (2019). Human-centered design lessons for implementation science: improving the implementation of a patient-centered care intervention. Jaids J Acquir Immune Defic Syndromes.

[17] Salloum RG, Shenkman EA, Louviere JJ, Chambers DA (2017). Application of discrete choice experiments to enhance stakeholder engagement as a strategy for advancing implementation: a systematic review. Implement Sci.

